# Identifying core habitats and corridors for giant pandas by combining multiscale random forest and connectivity analysis

**DOI:** 10.1002/ece3.8628

**Published:** 2022-02-14

**Authors:** Xue Sun, Zexu Long, Jingbo Jia

**Affiliations:** ^1^ 47820 College of Wildlife and Protected Area Northeast Forestry University Harbin China

**Keywords:** factorial least‐cost path, multiscale habitat selection, Qionglai mountain, random forest, resistant kernel, UNICOR

## Abstract

Habitat loss and fragmentation are widely acknowledged as the main driver of the decline of giant panda populations. The Chinese government has made great efforts to protect this charming species and has made remarkable achievements, such as population growth and habitat expansion. However, habitat fragmentation has not been reversed. Protecting giant pandas in a large spatial extent needs to identify core habitat patches and corridors connecting them. This study used an equal‐sampling multiscale random forest habitat model to predict a habitat suitability map for the giant panda. Then, we applied the resistant kernel method and factorial least‐cost path analysis to identify core habitats connected by panda dispersal and corridors among panda occurrences, respectively. Finally, we evaluated the effectiveness of current protected areas in representing core habitats and corridors. Our results showed high scale dependence of giant panda habitat selection. Giant pandas strongly respond to bamboo percentage and elevation at a relatively fine scale (1 km), whereas they respond to anthropogenic factors at a coarse scale (≥2 km). Dispersal ability has significant effects on core habitats extent and population fragmentation evaluation. Under medium and high dispersal ability scenarios (12,000 and 20,000 cost units), most giant panda habitats in the Qionglai mountain are predicted to be well connected by dispersal. The proportion of core habitats covered by protected areas varied between 38% and 43% under different dispersal ability scenarios, highlighting significant gaps in the protected area network. Similarly, only 43% of corridors that connect giant panda occurrences were protected. Our results can provide crucial information for conservation managers to develop wise strategies to safeguard the long‐term viability of the giant panda population.

## Graphical abstract

We combined an equal‐sampling multiscale random forest approach and connectivity modeling to predict habitat suitability and identify core habitats and corridors for the giant panda in Qionglai mountain. We assessed the effectiveness of protected areas in representing core habitats and corridors.

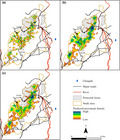

## 1 INTRODUCTION

The giant panda (*Ailuropoda melanoleuca*) is a rare and protected wildlife endemic to China and a flagship species of biodiversity conservation globally (Swaisgood et al., 2010). The giant panda once roamed throughout most of the lowlands of eastern and southern China, northern Vietnam, and northern Myanmar (Pan et al., 2014). Due to human activity, climate change, and natural disasters, the habitat of giant pandas has been continuously lost and fragmented. Currently, giant pandas are only distributed in part of mountainous areas in Sichuan, Shaanxi, and Gansu (State Forestry Administration, 2015). The Chinese government has made great efforts to protect this charming species by establishing 67 reserves, Grain to Green Project, and Natural Forest Protection Program (Wei et al., 2015). These conservation efforts have led to some achievements, including population growth and habitat expansion (State Forestry Administration, 2015). Results of the Fourth National Giant Panda Survey (hereinafter the fourth survey) revealed that there is an estimated population size of 1,864 individuals in the wild and showed a 16.8% population increase compared to the third survey, which was conducted from 1998 to 2001(State Forestry Administration, 2015). Based on the observed population increase, the International Union for Conservation of Nature (IUCN) changed the status of the giant panda from “endangered” to “vulnerable” (Swaisgood et al., 2016). However, the panda habitat is becoming increasingly fragmented (Xu et al., 2017), and small populations will face high extinction risks (Kong et al., 2021). According to the fourth survey, the panda's range is estimated to be subdivided into about 33 subpopulations separated by mountain ranges, rivers, roads, forest clearings, and human settlements (State Forestry Administration, 2015). Despite the enormous efforts that have been put in panda conservation, approximately 46% of the habitat (33% of the panda population) remains unprotected (State Forestry Administration, 2015). It is urgently needed for a knowledge‐based metapopulation management strategy for the long‐term viability of giant panda subpopulations (Wei et al., 2012). Establishing and protecting core habitat patches and the connectivity networks that connect them is one of the ways to ensure the long‐term survival of large terrestrial mammals at a regional scale (Kaszta et al., 2020). Additionally, it is unlikely to protect all the landscape with limited financial resources, large human populations, and complicated land ownership, making it crucial to identify core habitats and corridors. Several previous studies have identified habitat connectivity for giant pandas in different mountain ranges using least‐cost path analysis or circuit theory (Li et al., 2010; Qi et al., 2012; Wang et al., 2021). Researchers usually first mapped habitat patches based on habitat suitability and then simulated corridors among patches in these studies. There were some shortcomings in them. For example, it is a simplification using habitat patches as source points rather than species occurrences, besides few studies took giant panda dispersal ability into account, though these two aspects are foundations for reliable prediction of corridor networks (Cushman et al., 2012, 2013). Furthermore, it was hard to prioritize corridors in these assessments given corridor construction and restoration are projects that consume both huge manpower and money (Kang & Li, 2016). Therefore, there is a need to apply more comprehensive approaches to map corridors.

Landscape resistance is a crucial component in connectivity modeling. It is challenging to quantify resistance to movement in a large extent because movement data are usually unavailable. Given the lack of movement or genetic data, habitat suitability is frequently used as a proxy to reflect landscape resistance (Zeller et al., 2012). Therefore, habitat suitability models may have essential impacts on resistance estimation. Research on the habitat of giant pandas facilitates our understanding of the resource needs and ongoing threats and is also a necessary basis for conservation decision‐making (Hull et al., 2014). The habitat selection of animals is multidimensional, and the response of animals to different environmental factors often occurs at multiple hierarchical levels and over a range of spatial and temporal scales (Timm et al., 2016; Wiens, 1989). When describing the relationship between species and habitat, it is necessary to determine the suite of the covariates relevant to habitat selection by the species and determine the scale of interaction between species and habitat (Graf et al., 2005). Incorrect insight into the nature and significance of relationships between species responses and environmental variables may result from ignoring scale in habitat modeling (McGarigal et al., 2016). As an iconic species of global biodiversity conservation, the research on giant pandas and their habitats has received extensive attention (Bai et al., 2020; Hull et al., 2014, 2016; Wei et al., 2015). Surprisingly, almost all of these studies were conducted using a single‐scale model framework that all covariates are measured at the same spatial scale. These spatial scales are frequently determined arbitrarily by researchers or justified based on expert biological knowledge of the giant panda, such as 20 * 20 m plot size or 250 * 250 m raster cell size (e.g., Feng et al., 2009; Wang et al., 2010). Modeling habitat suitability under a multiscale modeling framework is superior to the single‐scale models in terms of model predictive ability and proportion of deviance explained for some species (Bellamy et al., 2013; Timm et al., 2016). In addition, the multiscale analysis provided new insight on the relationship between species response and habitat covariates that single‐scale model did not detect (Mateo‐Sánchez et al., 2014; Timm et al., 2016; Wasserman et al., 2012). Therefore, studying habitat selection using a multiscale framework is of significance for deepening our understanding of the relationship between giant pandas and their habitat and formulating more targeted conservation and management strategies.

For more than a decade, traditional statistical methods such as logistic regression have been the dominant method in multiscale habitat modeling (McGarigal et al., 2016). However, in ecological modeling, well‐defined issues, such as complicated nonlinear interactions, spatial autocorrelation, high‐dimensionality, nonstationarity, and scale, make it difficult for the collected ecological data to meet the assumptions of traditional statistical models (e.g., independence, homogeneity of variance, and multivariate normality), thereby reducing the robustness of the model results (Olden et al., 2008). In the face of these problems, the advantages of machine learning methods gradually emerge and are increasingly widely used in habitat modeling (Evans et al., 2011). Among them, the random forest model stands out because it can deal with a large number of predictors and find sound signals from data with noise. Random forest is an algorithm that developed out of classification and regression tree (CART) and bagging approaches (Breiman, 2001). It builds a classification and regression tree through repeated resampling to form a weak classifier and ensemble many weak classifiers to develop into a strong classifier. Many studies have shown that random forest outperforms traditional statistical methods in terms of model predictive ability (Cushman et al., 2017; Cushman & Wasserman, 2018; Mi et al., 2017), whereas there are few applications of random forest in giant panda habitat modeling, in which the most commonly used are maximum entropy model and habitat suitability index model (e.g., Songer et al., 2012; Xu et al., 2006).

This study tries to identify core habitats and corridors for giant pandas using the improved method by combining multiscale random forest habitat modeling and connectivity analysis. There are three specific objectives of this study: (1) we combine an extensive giant panda occurrence dataset and the multiscale random forest habitat modeling framework to delineate a habitat suitability map for giant pandas in the Qionglai mountain; (2) we used resistant kernel approaches and factorial least‐cost path analysis to identify core habitats and corridors; and (3) we assessed the representation of the predicted core habitats and corridors in the protected area network. Although using giant panda in the Qionglai mountain as a case study, the approaches are expected to provide crucial information for conservation managers to develop more effective conservation strategies for other wildlife species.

## 2 METHODS

### 2.1 Study area

**FIGURE 1:**
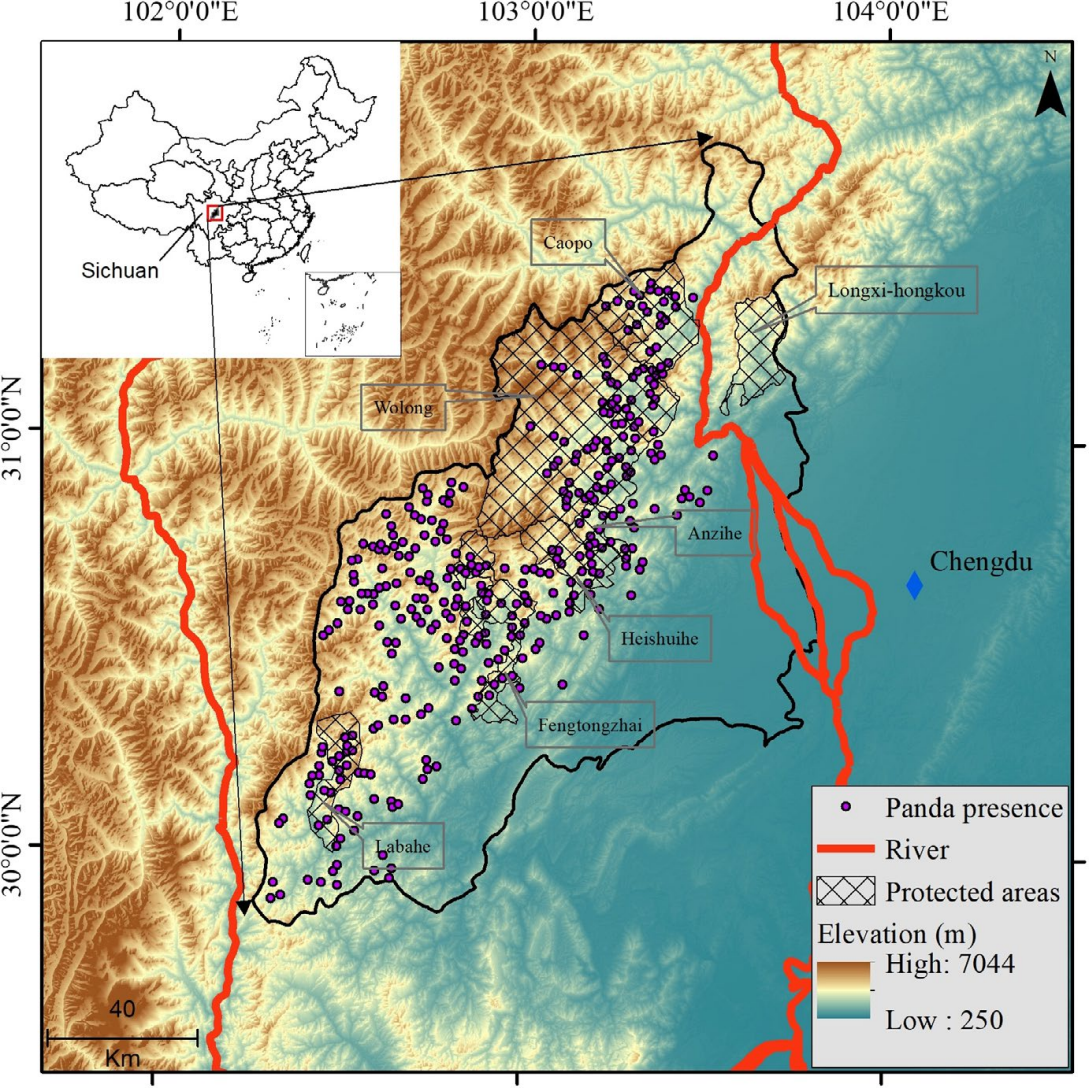
The geographic extent of the study area and distribution points of the giant panda in the Qionglai Mountain

The Qionglai mountain is located in the west of the Sichuan Basin and is the geographical boundary between Sichuan Basin and Tibetan Plateau. The study area comprises eight counties with a total area around 15,712 km^2^ (between 102.26° E and 103.82° E longitude and 29.82°N and 31.72°N latitude; Figure 1). There are six major vegetation/elevation zones in the Qionglai mountain range (Xu et al., 2006): (1) subtropical evergreen broad‐leaf forests below 1,600 m; (2) mixed forests of evergreen and deciduous broad‐leaf forests between 1,600 and 2,000 m; (3) coniferous and broad‐leaf mixed forests between 2,000 and 2,600 m; (4) subalpine coniferous forests between 2,600 and 3,600 m; (5) scrub meadows and alpine talus vegetation between 3,600 and 4,400 m; (6) screes and permanent snow belts above 4,400 m. Many rare wild animals coexist with giant pandas in the Qionglai mountain range, such as golden snub‐nosed monkey (*Phinopithecus roxellana*), red panda (*Ailurus fulgens*), sambar (*Cervus unicolor*), takin (*Budorcas taxicolor*), among others (Zhang et al., 2017).

The Qionglai mountain is where the giant panda was scientifically discovered (Hu, 2001). It also is the second‐largest tract of the habitat of the six mountain ranges occupied by giant pandas and is home to about 30% of the entire wild giant panda population (State Forestry Administration, 2015). The Qionglai panda population is estimated to be divided into five subpopulations, including Xiaojin, Wolong‐Caopo, Xiling‐Jiajin, Baishahe, and Sanhe, by major roads (e.g., G350, S210, G318) (Forestry Department of Sichuan Province, 2015). There are seven established protected areas in the study area (Figure 1), three of which are administrated at the national level: Wolong, Fengtongzhai, and Longxi‐Hongkou; the remaining four are at the provincial level: Anzihe, Heishuihe, Labahe, and Caopo.

### 2.2 Presence and pseudo‐absence points of giant pandas

The giant panda occurrence data used in this study were from the fourth survey conducted between 2011 and 2014 (Tang et al., 2015). Based on the third panda survey (1999–2003) results, the survey area in the fourth survey was classified into key survey areas (2 km^2^ survey cell size) and general survey areas (6 km^2^ survey cell size). In each survey cell, one line‐transect generally greater than 0.75 km was placed to cover all types of panda habitat. Walking along line transects, investigators collected evidence of giant panda occurrences, including feces, foraging sites, dens, footprints, or entities, and used GPS (Global Position System) to record the coordinates of these occurrences (Tang et al., 2015). To reduce the effect of spatial autocorrelation between occurrence points on model performance, we used “SDMtoolbox” to spatially filter panda occurrences (Brown & Anderson, 2014); the filter radius was set to 1.2 km according to the average home range size of giant pandas estimated based on GPS collar study in Wolong reserve (4.4 ± 1.2 km^2^; Hull et al., 2015). This means the minimum distance among the filtered occurrences is 1.2 km. Finally, 403 out of 528 giant panda occurrences were retained for modeling (Figure 1).

The survey results of giant pandas only include the occurrence points and do not include the absence data of giant pandas. In fact, due to the elusive behavior of giant pandas and dense vegetation, it is not practical to confirm the absence of giant pandas in a 2 km^2^ or 6 km^2^ survey cell (Viña et al., 2010). To apply the random forest model in the absence of reliable absence points, we generate a set of pseudo‐absence points based on the giant panda occurrence locations (Wang et al., 2010). To fully unleash the power of random forest, we used a random selection of geographically stratified pseudo‐absences approach recommended by (Barbet‐Massin et al., 2012). The pseudo‐absences should lie outside the 3‐km radius buffer zone (based on the maximum territory size of the giant panda, which is around 30 km^2^; Hu, 2001) of panda occurrences. The pseudo‐absences are also limited in areas with elevation <4,000 m and slope <50°as giant pandas often avoid high elevation and steep areas (Wang et al., 2010). The minimum distance among pseudo‐absences is also set to 1.2 km to alleviate spatial autocorrelation. We randomly select ten sets of the same number of pseudo‐absences as available presences (403 in this study) because random forest model is sensitive to class imbalance (Barbet‐Massin et al., 2012).

### 2.3 Environmental variables

According to previous studies, we selected a set of environmental variables that may affect giant panda habitat selection or distribution (Viña et al., 2010; Wang et al., 2010; Xu et al., 2006). These variables can be summarized into four categories: topographic, land cover, vegetation, and anthropogenic disturbance (Table 1).

**TABLE 1:** Predictor variables used in the analysis and their optimal scale identified by univariate random forest

Category	Variables	Description	Source	Optimal scale (km)
Topographic	ELE	Focal mean of elevation	NASA’S SRTM v4	1
	SLP	Slope position		5
	ASP	Slope aspect transformed to range 0–1 using methods in Roberts and Cooper (1989)		6
	TRI	Terrain ruggedness index		5
Vegetation	NPP	Net primary productivity	MODIS MOD17A3 product	1
	BAM	Percentage of bamboo coverage	Predicted from MaxEnt using MODIS phenological metrics	1
Land cover (Landscape level)	AI	Aggregation index for the full landscape mosaic within a moving window	FRAGSTATS analysis of the reclassified Copernicus land cover map	4
	ED	Edge density for the full landscape mosaic within a moving window		1
	PD	Patch density for the full landscape mosaic within a moving window		1
	SHDI	Shannon's diversity index for the full landscape mosaic within a moving window		2
Land cover (class level)	LPI_CNF	Largest patch index of the closed needle leaf forests within a moving window	FRAGSTATS analysis of the reclassified Copernicus land cover map	4
	PLAND_CNF	Percentage of the closed needle‐leaf forest within a moving window		2
	LPI_CBF	Largest patch index of the closed broad‐leaf forest within a moving window		1
	PLAND_CBF	Percentage of the closed broad‐leaf forest within a moving window		1
Anthropogenic	Disvil	Euclidean distance to the nearest village	1:250,000 National Basic Geographic Database	
	Dismajor	Euclidean distance to the nearest major road		
	Disunpaved	Euclidean distance to the nearest minor road		
	Densvil	The density of villages within a moving window		4
	Densrd	The density of all roads within a moving window		2

Topographic variables included elevation, degree of slope, aspect, and terrain ruggedness index. A 90‐m resolution digital elevation model (DEM) product was downloaded from the Shuttle Radar Topography Mission (SRTM; http://srtm.csi.cgiar.org). We calculated these four topographic variables using the Gradient and Geomorphometric Modeling Toolbox in ArcGIS (Evans et al., 2014). To avoid the circular issue of aspect, we transformed the aspect from the range 0–360 to the range 0–1 using the method developed by Roberts and Cooper (1989).

We obtained the land cover product from the Copernicus Global Land Service (Buchhorn et al., 2020), with a spatial resolution of 100 m (https://land.copernicus.eu/global/products/lc, 2015). The original land cover product has 22 categories which were reclassified into 7 categories including: crop, shrub, grass, closed broadleaf forest (canopy cover >70%; CBF), closed needle leaf forest (canopy cover >70%; CNF), open forest (canopy cover <70%; OF), and nonvegetation area. We then used FRAGSTATS v4.2 (McGarigal, 2002) to calculate four landscape‐level metrics (Aggregation Index, AI; Edge Density, ED; Patch Density, PD; Shannon Diversity Index, SHDI) and two class‐level metrics for the two dominant forest types (i.e., CNF and CBF) (Largest Patch Index, LPI_; Percentage of Landscape, PLAND_; Table 1) to characterize landscape composition and configuration.

**FIGURE A1:**
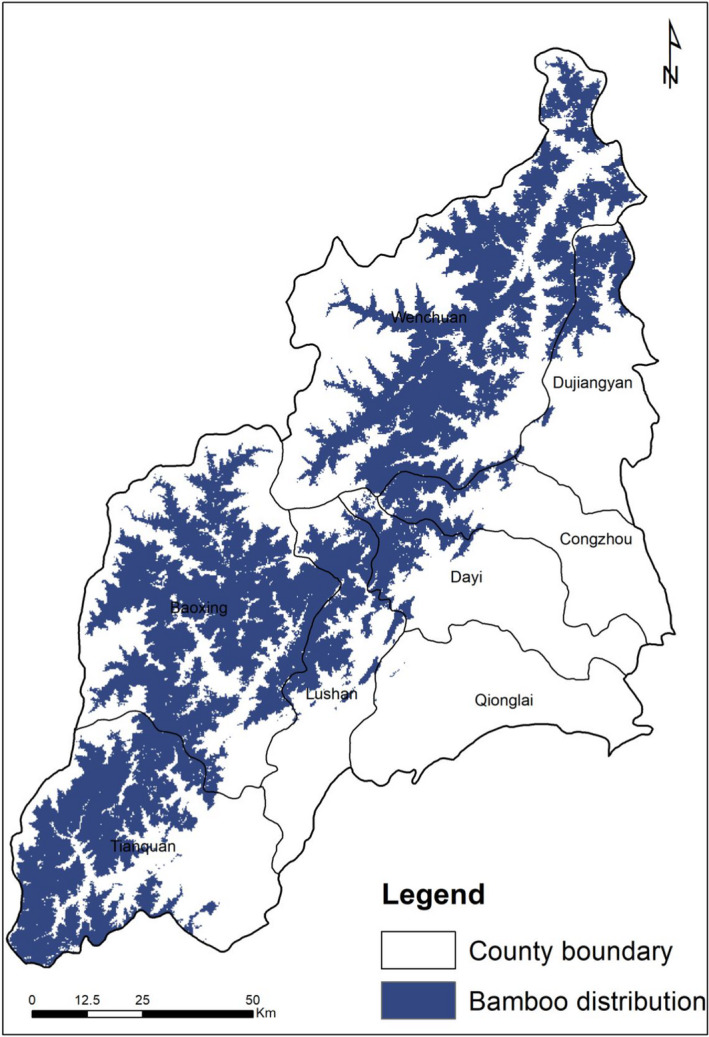
Binary map for bamboo distribution in Qionglai mountain predicted by using MaxEnt and phenological metrics

We used a remotely sensed measure of net primary productivity (NPP) obtained from the MODIS (Moderate Resolution Imaging Spectroradiometer) satellite image at 500‐m resolution (Running et al., 2004). We calculated a 4‐year average of NPP from 2011 to 2014 to be consistent with the time of the panda survey. We also included the bamboo distribution as a vegetation variable as the giant panda is primarily dependent on bamboo (Hu et al., 1985). Ground‐based surveys are unavailable for detailed information on bamboo's spatial distribution across large extents. Then, we modeled bamboo distribution using the method developed by Tuanmu et al. (2010). This method extracts 11 phenology metrics from a time series of MODIS satellite images and combines these metrics with the maximum entropy modeling (MaxEnt; a machine learning‐based species distribution model) to model the probability of bamboo presence. We used this method to model the probability of bamboo presence and converted it to a binary map of bamboo distribution (i.e., bamboo vs. no bamboo) using the threshold that maximizes the summation of model sensitivity and specificity (Liu et al., 2013). Finally, we calculated the proportion of bamboo coverage within a range of moving windows. See the bamboo modeling details in Appendix A and a binary distribution map for bamboo in Figure A1.

We calculated the Euclidean distance to the village, major road, and minor road for spatial measures of anthropogenic disturbance. We also calculated the density of villages and roads (major and minor) across various spatial scales. Shapefile of villages and roads were obtained from an open database from the National Basic Geographic Database (www.webmap.cn; 2015).

All variables were projected to the 48N UTM projection and resampled to a 250‐m spatial resolution in ArcGIS (ESRI, 2014). Categorical variables were resampled using the nearest neighborhood method, whereas continuous variables were resampled using the bilinear interpolation method.

### 2.4 Multiple scale variables

Scale optimization plays a vital role in habitat modeling (McGarigal et al., 2016). We transformed all variables but the distance‐based variables (i.e., distance to villages or roads) to multiple scale variables. We considered six spatial scales in the present study, including 1,000, 2,000, 3,000, 4,000, 5,000, 6,000 m; these scales correspond to a spatial extent of 3.14–113 km^2^, which include the average size of giant panda home range (Hull et al., 2015) and the minimum area requirements of a giant panda population (114.7 km^2^; Qing et al., 2016). Landscape metrics were calculated in FRAGSTATS (McGarigal, 2002) using the moving window option at the six spatial scales, while other variables were calculated of their focal mean at different radii using the “Multi‐scale Maxent Toolbox” in ArcGIS (Bellamy & Altringham, 2015).

### 2.5 Multiscale random forest habitat model

We used the random forest approach developed by Evans and Cushman (2009) to model habitat suitability for the giant panda in the Qionglai mountain. We conducted the random forests using the two‐step multiscale optimization framework suggested by McGarigal et al. (2016). First, for each presence and pseudo‐absence dataset, we run univariate random forest models to identify the optimized scale for each variable. Presence and pseudo‐absence points of giant pandas were used as response variables and were tested against one scale of each environmental variable at a time. The optimized scale of each variable was determined based on the model with the lowest out‐of‐bag (OOB) error rate. We used the scale with the highest frequency of selected optimal scale in the ten univariate random forest models as the final optimal scale for that variable. The scale‐optimized variables were further tested for multicollinearity and for those pairs with a Pearson correlation coefficient higher than 0.85, one variable was removed.

Second, utilizing the suite of scale‐optimized variables from the first step, we constructed a multivariate random forest model to predict the probability of giant panda occurrence. To identify the most parsimonious model, we used Model Improvement Ratio (MIR; Murphy et al., 2010) to retain only the most important variables. The MIR employs the permuted variable importance, represented by a decrease in OOB error standardized from zero to one. The variables are subset using 0.1 increments of MIR value in model selection, with all variables above the threshold retained for each model. This subset is always conducted on the original model's variable importance to avoid over‐fitting (Svetnik et al., 2004). We compared all subset models and selected the lowest total OOB error as the final model. Before any random forest modeling, we evaluated the minimum number of trees needed by evaluating 2,000 bootstrap samples and observed when the OBB error rate stopped improving. The result showed that OOB error rate ceased to improve after 200 trees (Figure A3), but we used 500 trees in all models to be conservative as Evans et al. (2011) recommended. Model building and selection were performed using the R package “rfUtilities” (Evans & Murphy, 2014) and “randomForest” (Liaw & Wiener, 2002). Model predictions for the random forest model were generated by creating a habitat suitability map using a ratio of the majority in the votes matrix. We repeated the above steps for the ten presence and pseudo‐absence datasets. The final habitat suitability prediction is averaged over the ten models (Barbet‐Massin et al., 2012; Valavi et al., 2021); this method is called equal‐sampling random forest. We also plotted the partial plots for the selected variables by plotting the range of a variable against the estimated probability while keeping other variables at their mean.

### 2.6 Model evaluation

We assessed the predictive performance of the equal‐sampling multiscale random forest model using the area under the total operating characteristic curve (AUC). AUC is a threshold‐independent evaluation metrics, it measures the ability of the model to discriminate presences from pseudo‐absences (Pearce & Ferrier, 2000). Presences and pseudo‐absences of giant pandas were randomly divided into a training set (70%) and a validation set (30%). This procedure was repeated ten times, and we calculated the mean AUC.

### 2.7 Landscape resistance layer

A study has shown that the relationship between the resistance species moving in the landscape and the habitat suitability is usually an exponential function rather than a linear function (Keeley et al., 2016). We converted the predicted habitat suitability map from multiscale random forest to the landscape resistance layer using an exponential function (Equation 1):

(1) $$R=1000^{\left(‐1*\text{HS}\right)}$$

where *R* is the resistance value, and HS is the predicted habitat suitability. We then rescaled the resistance values to the range between 1 and 100 using linear interpolation, such that the resistance values equal 1 when HS is 1 and 100 when HS is 0. Such transformation means that most pixels in the studied landscape receive low resistance values, and only areas with very low habitat suitability receive high resistance values (Keeley et al., 2016).

### 2.8 Identification of core habitats and corridors

We used the resistant kernel method (Compton et al., 2007) and factorial least‐cost path analysis (Cushman et al., 2009) in the universal corridor network simulator (UNICOR) (Landguth et al., 2012) to create two connectivity predictions: resistant kernels and factorial least‐cost paths. The resistant kernel method calculates the cumulative resistance cost‐weighted dispersal kernel around each source point up to a threshold (usually determined by species movement ability), then summing all kernels to create a surface of expected density of dispersing organisms at any location in the landscape (Compton et al., 2007). This surface is a function of source points, landscape resistance, and dispersal ability (Cushman, McRae, et al., 2013). The factorial least‐cost path analysis uses Dijkastra's algorithm to calculate the least‐cost path from every species occurrence to every other occurrence location in the landscape (Landguth et al., 2012). These simulated least‐cost paths were then buffered based on kernel density estimation, and the Gaussian function was selected in our study. All buffered least‐cost paths were summed to produce a map of corridor intensity. The value of a pixel in this map represents the frequency of least‐cost paths passing through it.

We used the spatially filtered giant panda occurrences as source points and the transformed habitat suitability map as the resistance layer. A previous study demonstrated that dispersal ability has a significant effect on the estimation of population connectivity (Cushman et al., 2013). However, we do not have a certain knowledge of giant pandas’ dispersal ability. To account for uncertainties regarding giant panda dispersal ability, we used three distance thresholds in the resistant kernel analysis: 6,000, 12,000, 20,000 cost units, which indicate movement distance of 6, 12, 20 km, respectively, in ideal low resistance habitat. We selected 6 km because the biggest home range recorded was about 30 km^2^ (Hu, 2001); if the home range was seen as a circle, its diameter is approaching 6 km. In addition, genetic studies indicated that the spatial extent of the genetic structure of one population occurred within about 12 km (Hu et al., 2010; Zhan et al., 2007). Furthermore, Pan et al. (2014) and Zhan et al. (2007) reported several giant panda dispersal events with a distance exceeding 20 km. For factorial least‐cost paths analysis, we set the upper limit of dispersal ability to 50,000 cost units to model long‐distance connections, as the maximum dispersal distance recorded was 50 km (Swaisgood et al., 2010). We used the resistant kernel maps to identify core habitats for the giant panda, as in Cushman, Landguth, et al. (2013). We defined core habitats as contiguous areas with resistant kernel values greater than 5% of the highest value.

### 2.9 Effectiveness of protected areas

To assess the effectiveness of the current protected area network on protecting core habitat patches and corridors for the giant pandas in the Qionglai mountain, we quantified the extent and proportion of predicted core habitats and corridors within protected areas.

## 3 RESULTS

### 3.1 Scale optimization

**FIGURE A2:**
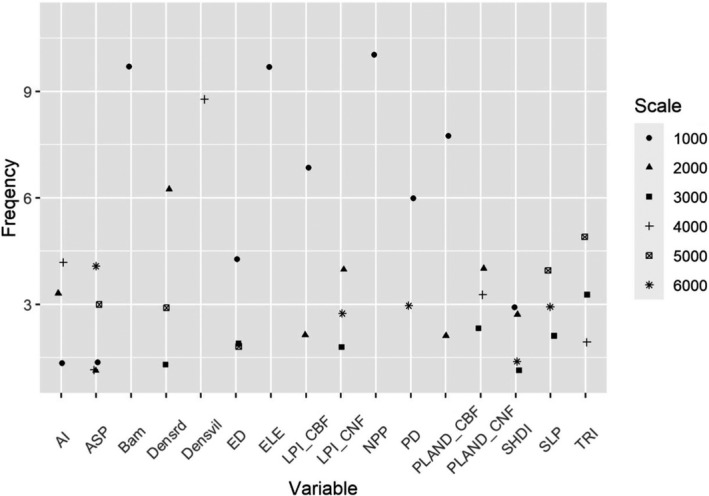
The frequency scale was selected as the optimal scale in 10 univariate equal‐sampling random forest models for each variable

**FIGURE A3:**
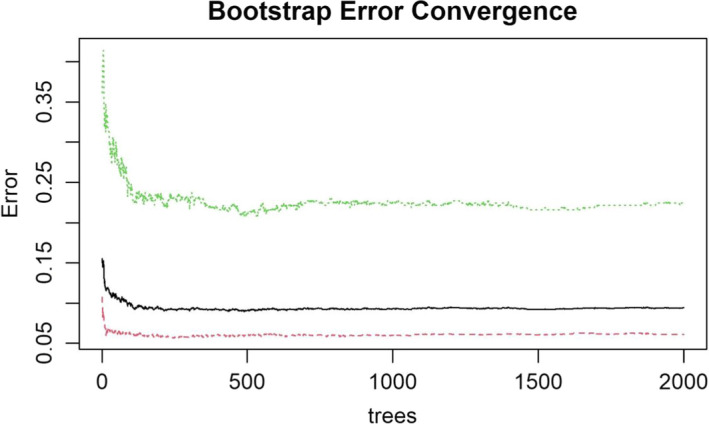
The number of trees versus the error rate plot

The univariate random forest optimization analysis showed that optimized scales vary by variables (Table 1; Figure A2); bamboo cover, elevation, net primary productivity, patch density, and largest patch index of closed broad‐leaf forest were all strongly related to giant panda occurrence at fine scale (1,000 m), while aspect, terrain ruggedness index, village density, percentage of closed needle‐leaf forest were strongly related at much coarser scale (≥4,000 m). Four variables (PLAND_CBF, ED, PLAND_CNF, SLP) were screened due to their high correlation with other variables.

### 3.2 Multivariate random forest model

**FIGURE A4:**
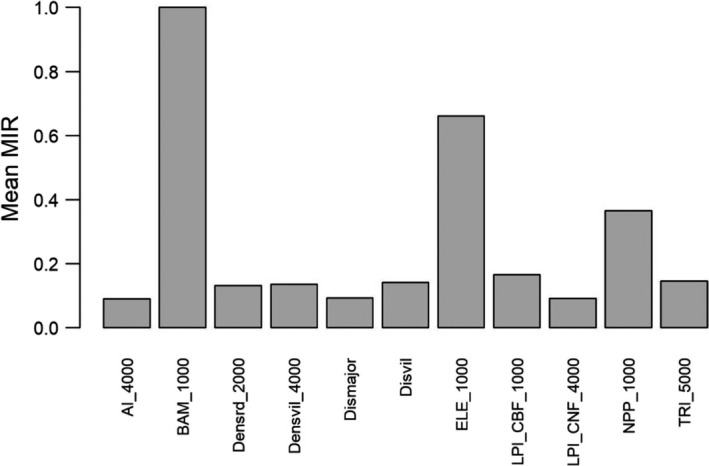
Bar plot of variable importance based on the mean Model Improvement Ratio (MIR) from random forests of 10 equal‐sampling presence‐pseudo absence datasets

We selected the most parsimonious model based on MIR for each presence and pseudo‐absence dataset. The most significant variables were the percentage of bamboo cover, elevation, and net primary productivity, while other variables had a relatively low influence on giant panda occurrence (Figure A4).

**FIGURE 2:**
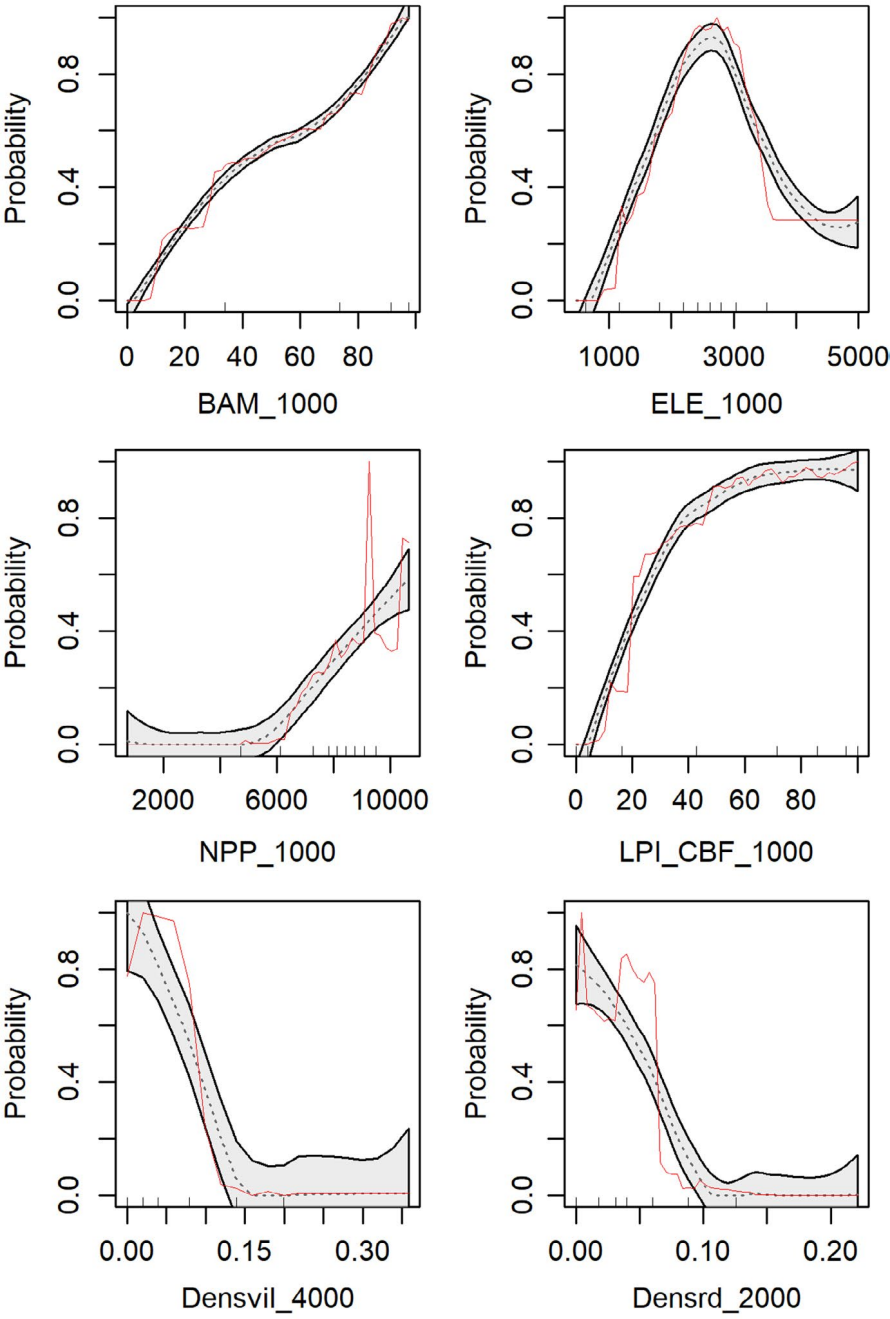
Partial dependency plots representing the marginal effect of habitat variables on predicted occurrence of giant panda. The gray area indicates the 95% confidence interval, and the red line indicates the mean average

Our equal‐sampling multivariate random forest model showed that predicted giant panda occurrence probability had a nonlinear relationship with most variables while had an approximately linear relationship with the percentage of bamboo and the largest patch index of closed broadleaf forest (Figure 2). Elevation showed a unimodal relationship with giant panda occurrence probability, peaking at 2,600 m. Percentage of bamboo cover, largest patch index of CBF, and net primary productivity showed a positive association with giant panda occurrence. In contrast, village density and road density showed a negative relationship.

**FIGURE 3:**
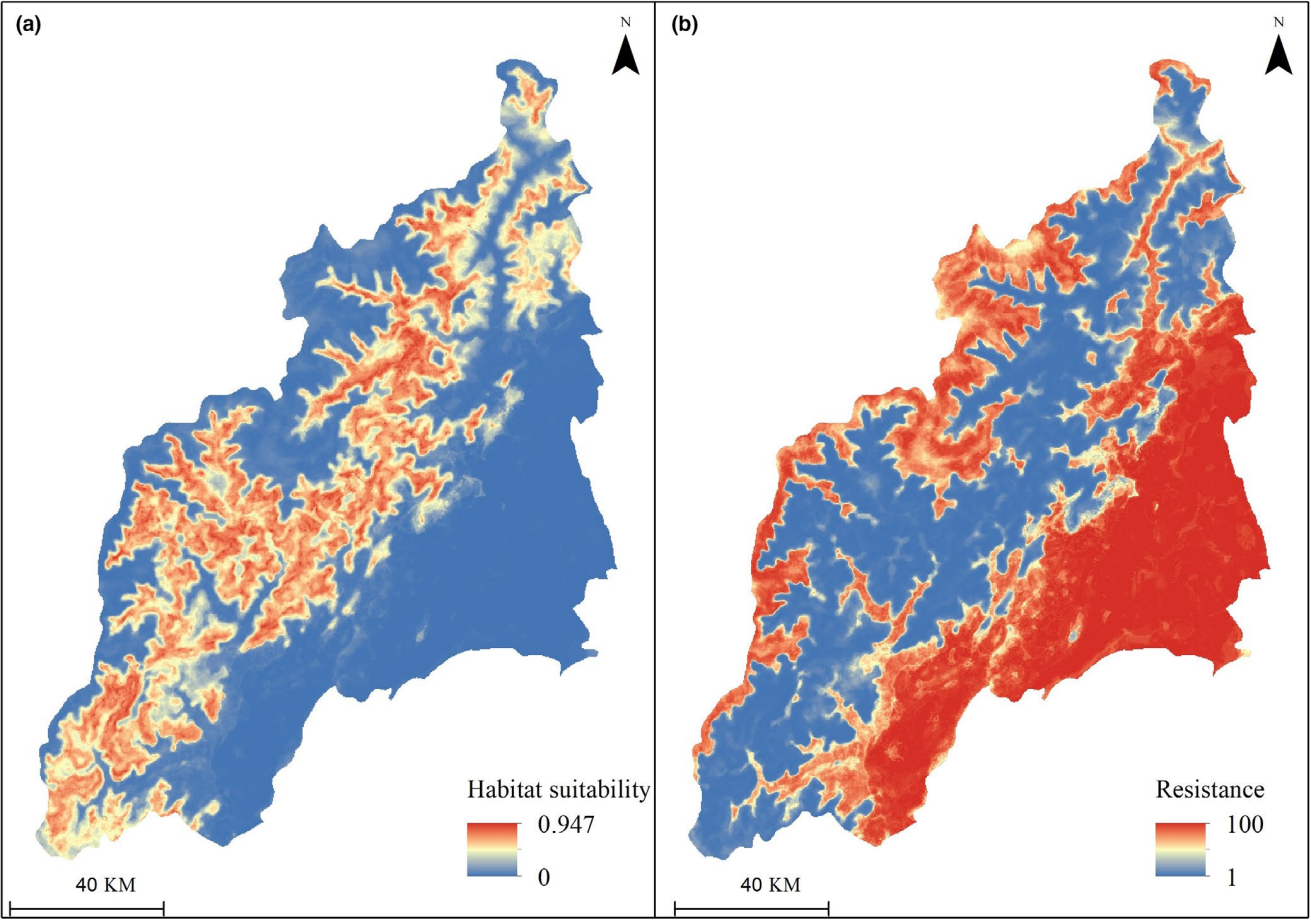
(a) The habitat suitability map shows giant panda's predicted occurrence based on equal‐sampling multiscale random forest habitat modeling in the Qionglai mountain. (b) The landscape resistance map shows the movement resistance for the giant panda, which is transformed from habitat suitability using an exponential function

The equal‐sampling multiscale random forest model showed an excellent predictive performance with a mean AUC value of 0.941 (SD = 0.014). The habitat suitability map (Figure 3a) produced by averaging ten predictions showing the predicted occurrence of giant pandas in the Qionglai mountain. Areas of low resistance to giant panda movement were concentrated mainly in the mid‐elevational part of the landscape (Figure 3b). Areas of high resistance were either in low elevation areas dominated by anthropogenic disturbance such as farmlands or in higher elevation areas where massive energy was required for giant pandas to move.

### 3.3 Core habitats and corridor network

**FIGURE 4:**
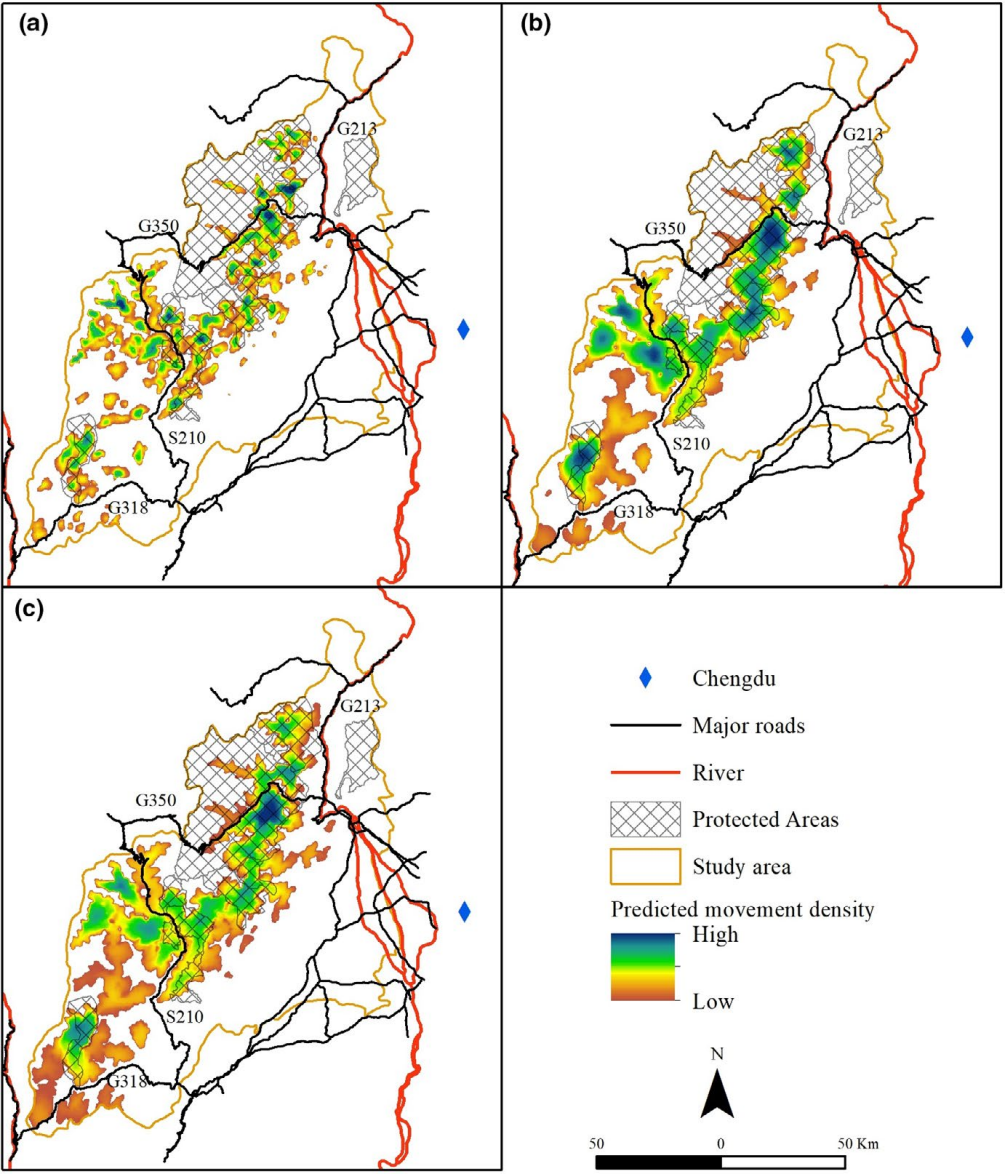
Resistant kernel value gradient for core habitat under different dispersal ability scenarios: (a) 6,000, (b) 12,000, and (c) 20,000 cost units

We presented resistant kernel simulation results for the giant panda in the Qionglai mountain at three dispersal scenarios (i.e., 6,000, 12,000, 20,000 cost units; Figure 4). Our connectivity simulation showed that high predicted rates of panda movement were mainly concentrated in the northern and central parts of the study area and a relatively small area in the southern region. Dispersal ability showed a significant effect on population connectivity simulation for the giant panda, with a broader range of connected area produced at high dispersal ability scenario (20,000 cost units; Figure 4c) than at low dispersal ability scenario (6,000 cost units; Figure 4a). Under the 6,000 cost units scenario, the giant panda population in the Qionglai mountain was predicted to be broken up into more than ten core patches (three large patches and several small patches; Figure 4a). Under the 12,000 cost units scenario, there were predicted to be two large patches and a few small patches (Figure 4b). Under the highest dispersal ability scenario (i.e., 20,000 cost units), most of the giant panda population was predicted to be connected within one dominant patch while a few small patches were isolated (Figure 4c). However, patches on each side of G318 could not be connected under all dispersal scenarios.

The extent of predicted core habitats varied between 3,451 km^2^ and 5,450 km^2^ along with dispersal ability, with more remarkable dispersal ability indicating a more significant predicted core habitats extent (Table 2).

**TABLE 2:** The extent and percentage of predicted core habitats covered by protected areas for the giant panda in the Qionglai mountain

Dispersal threshold (cost units)	Extent of core habitats (km^2^)	Extent of protected core habitats (km^2^)	% of protected core habitats
6,000	3,451	1,485	43%
12,000	4,648	1,853	40%
20,000	5,450	2,074	38%

The extent and percentage of protected core habitats differed along with dispersal ability scenarios. The area of protected core habitats varied between 1,485 km^2^ and 2,074 km^2^, with the protection rate ranging between 43% and 38% (Table 2).

**FIGURE 5:**
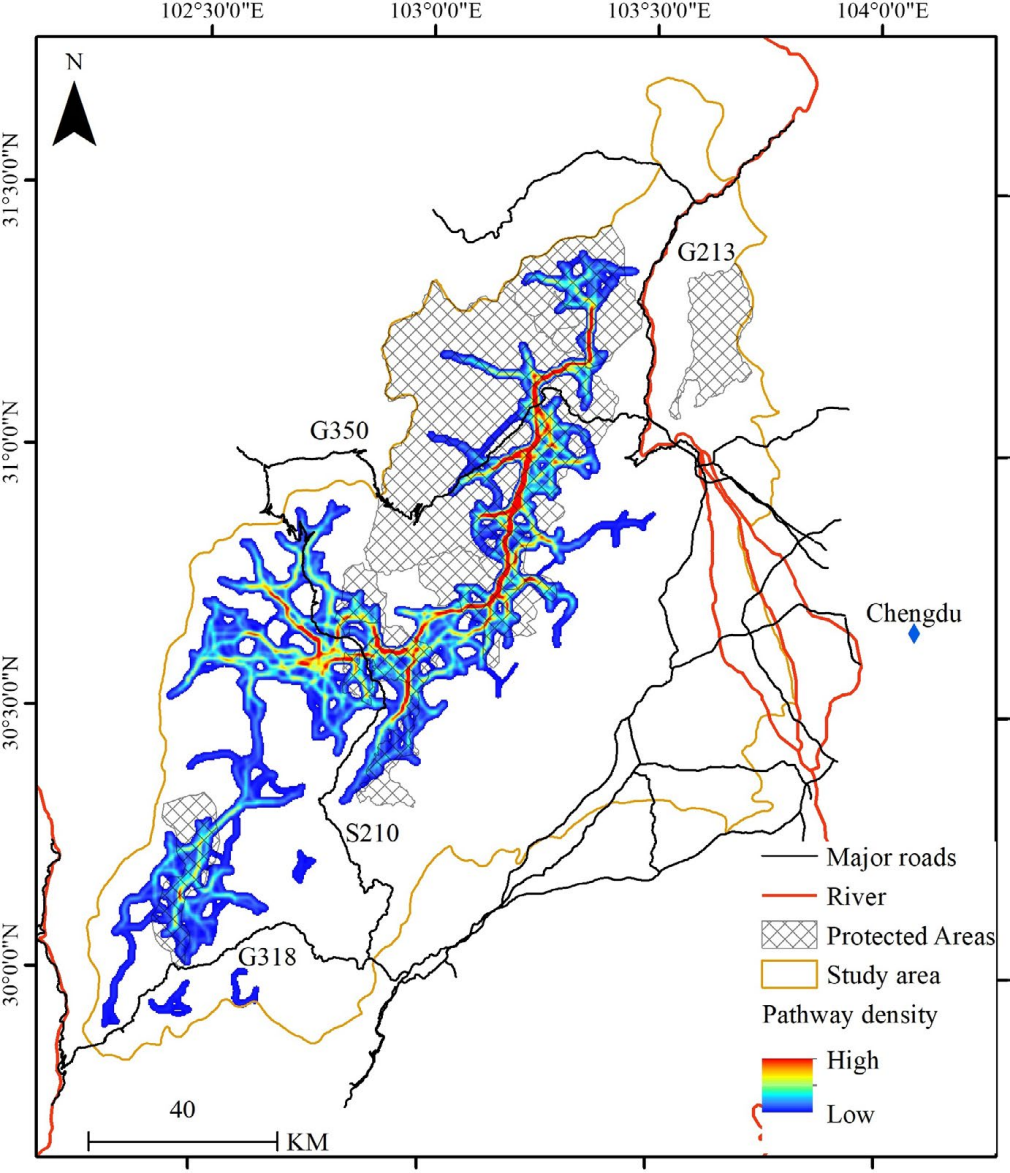
Corridor pathway density for the giant panda in the Qionglai mountain calculated by factorial least‐cost paths analysis under a dispersal threshold of 50,000 cost units. Corridor pathway density was shown with a gradient from weak (blue) to strong (red)

The factorial least‐cost paths map (Figure 5) showed that dominant pathway density lies in the mountain area's northern and central parts. The extent of simulated corridors was 3,234 km^2^, of which protected areas covered 1,394 km^2^ (43%).

## 4 DISCUSSION

This study presents one example of a scale‐optimized habitat selection model and the only example we are aware of for the endangered giant panda at a mountain extent. We quantitatively assessed population connectivity for the giant panda in the Qionglai mountain, combining the species distribution and connectivity modeling technique. The approach applied in this study can be used to identify primary resource requirement, limiting factors, and the spatial scales that giant panda strongly associated with habitat components. Our results provide crucial information to assist giant panda conservation management, including identifying the distribution and strength of core habitat and areas as corridors that facilitate connectivity among core habitats.

### 4.1 Scale dependence of habitat selection

Scale is a vital component to consider in ecological research, and grain size is one of its key facets (Connor et al., 2018). Connor et al. (2018) showed that predictor grain size significantly impacts species distribution model accuracy and area of species presence prediction. But there are differences between their study and ours. They constructed species distribution models using variables that are all calculated within the same size moving window (i.e., same spatial scale) at one time (Connor et al., 2018). Strictly, their study should be considered as multiple single‐scale models rather than multiscale models because in the latter different variables can be included in the final model at variable scales (McGarigal et al., 2016). The missing step between them is scale optimization. Scale optimization is critical for robust habitat models, which is crucial in developing conservation and management strategies for endangered species (Timm et al., 2016) like giant pandas. A few studies have demonstrated that multiscale habitat models can improve model performance and deepen our understanding of the relationship between species and habitat (Mateo‐Sánchez et al., 2014; Timm et al., 2016). For example, including a variable in a habitat model with an inapposite scale may result in misleading variable importance (Connor et al., 2018).

Consistent with other studies on habitat selection of carnivores (Khosravi et al., 2019; Macdonald et al., 2019; Mateo‐Sánchez et al., 2014), the giant panda in Qionglai select different resources at varying spatial scales. Giant pandas respond to village density strongly at a broad scale (4 km), which highlights the importance of a large extent of the undisturbed landscape. On the contrary, our result revealed that giant pandas select bamboo cover proportion at a relatively fine scale (1 km). These findings were consistent with other studies on giant panda habitat selection, which concluded that pandas select for the disturbance at the level of geographic range and select for bamboo at the level of home range (Hull et al., 2014).

### 4.2 The effect of predictors on the distribution of giant pandas

We used an equal‐sampling multiscale random forest habitat modeling framework to delineate the relative habitat suitability map for the giant panda in the Qionglai mountain. Random forest is a tree‐based method based on “bagging” and is demonstrated to outperform traditional statistical models in the field of species distribution models (Cushman et al., 2017; Evans et al., 2011). In addition, the habitat suitability map derived from random forests is more discriminatory, with higher spatial heterogeneity than predictions from traditional generalized linear models like logistic regression (Cushman & Wasserman, 2018), providing a more robust delineation of priority suitable areas. Different from the general implementation of random forest in modeling species spatial distribution (Cushman & Wasserman, 2018; Dar et al., 2021; Rather et al., 2020) that only randomly select one set of pseudo‐absence sample of size equal to the number of presences, in this study, we used the equal‐sampling method. The idea of equal‐sampling is to fit *n* different random forest models (where *n* is often 10) on *n* different pseudo‐absence samples of size equal to the presences (Barbet‐Massin et al., 2012). This method is demonstrated suitable for presence‐background data and outperforms other random forest implementations (weighting or regression; Valavi, Elith, et al., 2021) and other modeling methods (e.g., generalized linear model and generalized additive model; Valavi et al., 2021).

As expected, the percentage of bamboo cover is the most crucial predictor driving the distribution of giant pandas. The giant panda is a specialist species with bamboo comprising about 99% of its diet, and it may spend up to 14 h/day foraging bamboo (Schaller, 1985). Therefore, including biotic interaction with bamboo will improve the performance of habitat models of the giant panda. Modeling understory bamboo distribution in dense forests to a large extent is challenging; however, phenological variables derived from time‐series remote sensing images (e.g., MODIS) provide a way to address such an issue (Tuanmu et al., 2010).

We found that road density and village density had more significant impacts on the giant panda occurrence probability than the distance to road or village. This finding indicated panda's relative habitat suitability is more related to human activities in the landscape than the proximity to linear roads. Human activity is the primary deterrent to giant pandas’ road use; sometimes, low‐use roads such as abandoned logging roads were positively related to panda's habitat selection (Qi et al., 2011).

The largest patch index of closed needle forest (LPI_CBF) was positively associated with panda occurrence probability, highlighting that pandas prefer large dense forest patches, a result similar to the result of Wang et al. (2010). Of the selected variables, the landscape composition variables (i.e., LPI_CBF_1000 and LPI_CNF_4000) were more important than the variables reflecting landscape configuration (i.e., AI_4000). This result is similar to other studies on Ursidae (e.g., brown bear; Mateo‐Sánchez et al., 2014) and is in agreement with the general pattern that habitat extent is more important than habitat configuration (Cushman & McGarigal, 2002). The giant panda has a high dependence on forest cover and has poor movement ability, suggesting that habitat composition should dominate its habitat relationships.

### 4.3 Giant panda population connectivity and corridors

Through many years of protection, the population number and habitats of wild giant pandas have increased (State Forestry Administration, 2015), significant species conservation results have been achieved in China (Kang & Li, 2016). However, habitat fragmentation has always been the key factor threatening their survival and is getting worse (Xu et al., 2017). A few studies assessed population connectivity and proposed corridors to link fragmented habitat patches (Li et al., 2010; Qi et al., 2012; Wang et al., 2021). The method usually used in these studies was the least‐cost analysis (Li et al., 2010; Qi et al., 2012), which simulated narrow linear corridors and the structural connectivity among habitat patches. Giant pandas may not use those simulated corridors because there may be no individuals in predicted habitat patches or giant pandas are hard to traverse long corridors due to limited mobility. We integrated panda's dispersal ability into connectivity analysis, which previous studies usually ignore. One strength of the resistant kernel approach is its explicit and realistic incorporation of species dispersal ability (Landguth et al., 2012). There are predicted to be >10 core habitat patches if the dispersal ability of giant panda is limited to 6,000 cost units, but with the dispersal of 12,000–20,000 cost units, it would result in seven to four patches (Figure 4). This result highlights that the extent and fragmentation of connected habitats are highly dependent on the dispersal ability of the focal species (also see Ashrafzadeh et al., 2020; Cushman, Landguth, et al., 2013). In this present study, under all dispersal ability scenarios, panda populations in Caopo, Wolong, Anzihe, Heishuihe, and Fengtongzhai were predicted to be connected by dispersal. However, population connectivity evaluation based on major roads divided this population into two subpopulations: the northern Wolong‐Caopo subpopulation and southern Xiling‐Jiajin subpopulation (State Forestry Administration, 2015). Functional population connectivity is a complex interaction between dispersal ability, population size, and resistance to movement (Cushman et al., 2010). Delineating subpopulations based on habitat patterns may underestimate population connectivity. For example, in the study of Xu et al. (2006), national and provincial roads (G318, G350, and S210) divided giant panda population in the Qionglai mountain into four blocks. However, in our estimation, G350 and S210 did not completely separate the big population into different subpopulations, which means giant pandas may cross these roads. Our results were supported by a recent large genetic study conducted in Wolong reserve (Qiao et al., 2019); Qiao et al. (2019) found no significant genetic boundaries exists within panda population despite the national road G350 that bisects the Wolong nature reserve. They recorded four giant panda road‐crossing events within 1 year, indicating giant panda populations may be better connected than previously thought.

Although dispersal ability significantly affects population connectivity, our evaluation was not based on the certain knowledge of giant panda dispersal ability due to lacking empirical study of giant panda movement in the real landscape. Future research using satellite tracking methods such as GPS collars will strengthen our understanding of the movement and dispersal of giant pandas, eventually improving the assessment of population connectivity. Our resistant kernel estimation highlights the distribution of connected habitats, and the resistant kernel value can potentially be used to prioritize areas for conservation that maximally protect the total connectivity of the population. Conservation practitioners can use such spatial‐explicit information to develop landscape conservation strategies when ecological, economic, and social constraints exist and priority areas should be planned (Kang & Li, 2016). Furthermore, spatial‐explicit resistant kernel estimation can provide more information in the zoning or the effectiveness evaluation of protected areas (Cushman et al., 2012) than simple habitat suitability distribution map, which is often used in such assessments (Qi et al., 2015; Wang et al., 2021). Under all dispersal ability scenarios, there is predicted to be a large proportion (57%–62%) of core habitats that are not protected by the current nature reserve network (Figure 4), highlighting a great potential to establish new protected areas.

The factorial least‐cost path analysis identified optimal routes between giant panda occurrences to facilitate connectivity. General corridor simulation methods (e.g., least‐cost path or least‐cost corridor) take habitat patches as the “source” and then calculate the path with the least cumulative cost between source patches; its result only reveals the location of the corridor (Cushman, McRae, et al., 2013). However, the factorial least‐cost path approach simulates corridor network based on species occurrence, and the simulation result provides the location and intensity of the corridor. The corridor intensity is a kernel density estimation based on the number of least‐cost paths. Such information is important for corridor priority planning as corridor building and restoration usually need to invest a lot of money and manpower. We recommend paying more attention to the corridors linking small populations with much higher extinction risk to large populations. For example, subpopulations at the southernmost of the study area need more attention as they are predicted to be isolated from large core habitat patches under all dispersal ability scenarios. In previous evaluations, these subpopulations were also believed to be isolated from other subpopulations by national roads (G318) (State Forestry Administration, 2015; Xu et al., 2006). In addition, areas with high predicted least‐cost paths frequency outside protected areas also need prior protection. We can identify barriers that may impede giant panda dispersal based on the corridor pathway, like major roads. Combined with corridor density, we can further locate and rank the intersection of corridors with roads, which can provide crucial information for conservation practitioners to implement road mitigation measures such as warning signs, reduced speed limits, fencing, and construction of crossing structures (Cushman, Lewis, et al., 2013; Zeller et al., 2020).

### 4.4 Caveats and limitations

Our findings must be interpreted with regard to several major considerations. First, giant panda occurrences used in this study were from the fourth survey conducted between 2011 and 2014. Natural and socio‐economical conditions have changed since then (Xu et al., 2017). For example, the reduction of total and agricultural population and the increasing of infrastructure development (e.g., hydro‐power stations and road construction). Therefore, care should be taken when interpreting our results. While our study may not provide robust support for current decisions because it is based on older data, it illustrated a way to identify core habitats and corridors for large terrestrial mammals. Second, other human disturbances (e.g., livestock and trails) negatively affect giant panda habitat suitability and were not included in the habitat modeling, so our study may have overestimated habitat suitability in some areas. Third, it should also be noted that habitat suitability is not a good proxy for landscape resistance, as habitat suitability reflects habitat selection in home range while species may use the landscape differently during dispersal movements (Keeley et al., 2017). It would be better to develop resistance models with movement (Zeller et al., 2018) or gene flow (Cushman et al., 2006) data. Lastly, when designing new protected areas or corridors, it is also necessary to consider other large carnivores, as long‐term monitoring studies have revealed a wide distribution range retreat of large carnivore populations across the giant panda distribution range (Li et al., 2020).

## CONFLICT OF INTEREST

The authors declare that they have no conflict of interest.

## AUTHOR CONTRIBUTIONS

**Xue Sun:** Conceptualization (lead); data curation (equal); formal analysis (equal); methodology (equal); writing – original draft (lead). **Zexu Long:** Conceptualization (equal); formal analysis (equal); methodology (lead); software (equal); visualization (equal); writing – review and editing (equal). **Jingbo Jia:** Data curation (equal); funding acquisition (equal); resources (lead); supervision (equal); writing – review and editing (equal).

## Data Availability

Environmental variables and R scripts used to generate the results presented in this study can be found at Figshare: https://doi.org/10.6084/m9.figshare.18482636.

## APPENDIX A

### Methods to model bamboo distribution

Bamboo distribution and abundance are crucial factors shaping giant pandas’ distribution as bamboo comprises about 99% of their diet (Schaller, 1985). Previous studies have demonstrated that the vegetation phenology derived from the moderate resolution imaging spectroradiometer (MODIS) satellite images can map the distribution of understory bamboo (Tuanmu et al., 2010; Viña et al., 2010). We obtained the time‐series MODIS imagery (eight‐day L3 Global 250m product, MOD09Q1) between 2011 and 2013. We then calculated the wide dynamic range vegetation index (WDRVI) (Gitelson, 2004) for each eight‐day image. The WDRVI is more suitable than NDVI for detecting the phonologic change in areas with high vegetation biomass, such as the forests with dense understory bamboo in our study area (Tuanmu et al., 2010). We then used TIMESAT 3.3 (Jönsson & Eklundh, 2004) to extract annual dynamic curves of the WDRVI. From the fitted WDRVI dynamic curves, we obtained 11 phenology metrics for each year, including the start‐of‐season time, end‐of‐season time, date of middle season, length of season, base level, maximum level, amplitude, large integral, small integral, increase rate, and decrease rate (Eklundh & Jönsson, 2012; Tuanmu et al., 2010; Viña et al., 2010). The 3‐year averages of these phenology metrics from 2011 to 2013 were used to map bamboo distribution. In addition, we included the elevation as a variable for modeling bamboo distribution because vegetation type is closely related to elevation zones in the Qionglai mountain range (Xu et al., 2006). We used the software Maxent (version 3.4.1, Phillips et al., 2006) to build a model for mapping the overall bamboo distribution as in Tuanmu et al. (2010). We did not have random bamboo presence localities across the study area, and we used two types of points as surrogate bamboo occurrence. One is the giant panda occurrence localities associated with bamboo from the Fourth Giant Panda Survey in Sichuan Province. The other is 500 points randomly selected from a coarse bamboo distribution map obtained from the 4th National Giant Panda Survey. We randomly selected 75% of occurrence data as the training dataset, and the remaining 25% were used as the validation dataset. The procedure was repeated ten times to get an average prediction of bamboo occurrence probability. We set other parameters in Maxent as default. To get a binary map of bamboo distribution, we used the threshold that maximizes the sum of sensitivity and specificity to convert the continuous occurrence probability map into a binary map (Liu et al., 2013).
